# Level of asthma control and its relationship with medication use in asthma patients in Brazil*

**DOI:** 10.1590/S1806-37132014000500004

**Published:** 2014

**Authors:** Josiane Marchioro, Mariana Rodrigues Gazzotti, Oliver Augusto Nascimento, Federico Montealegre, James Fish, José Roberto Jardim

**Affiliations:** Federal University of São Paulo Paulista School of Medicine, São Paulo, Brazil; Federal University of São Paulo Paulista School of Medicine, São Paulo, Brazil; Federal University of São Paulo Paulista School of Medicine, São Paulo, Brazil; Merck, Sharp & Dohme Corp., São Paulo, Brazil; Merck, Sharp & Dohme Corp., São Paulo, Brazil; Federal University of São Paulo Paulista School of Medicine, São Paulo, Brazil

**Keywords:** Asthma/therapy, Asthma/prevention and control, Medication adherence

## Abstract

**OBJECTIVE:**

To assess asthma patients in Brazil in terms of the level of asthma control, compliance with maintenance treatment, and the use of rescue medication.

**METHODS:**

We used data from a Latin American survey of a total of 400 asthma patients in four Brazilian state capitals, all of whom completed a questionnaire regarding asthma control and treatment.

**RESULTS:**

In that sample, the prevalence of asthma was 8.8%. Among the 400 patients studied, asthma was classified, in accordance with the Global Initiative for Asthma criteria, as controlled, partially controlled, and uncontrolled in 37 (9.3%), 226 (56.5%), and 137 (34.3%), respectively. In those three groups, the proportion of patients on maintenance therapy in the past four weeks was 5.4%, 19.9%, and 41.6%, respectively. The use of rescue medication was significantly more common in the uncontrolled asthma group (86.9%; p < 0.001).

**CONCLUSIONS:**

Our findings suggest that, in accordance with the established international criteria, asthma is uncontrolled in the vast majority of asthma patients in Brazil. Maintenance medications are still underutilized in Brazil, and patients with partially controlled or uncontrolled asthma are more likely to use rescue medications and oral corticosteroids.

## Introduction

Asthma is a worldwide, common chronic disease that affects individuals of all ages and has a great influence on patient quality of life. ^(^ 1 ^,^ 2 ^) ^In 2006, the International Study of Asthma and Allergies in Childhood reported that, in Brazil, the prevalence of asthma was 24.3% in schoolchildren and 19.0% in adolescents,^(^ 3 ^,^ 4 ^)^ whereas the prevalence of physician-diagnosed asthma was approximately 10%. Because of its high prevalence, asthma has a major socioeconomic impact,^(^ 5 ^)^ given that, when it is uncontrolled, it can lead to hospitalizations and school/work absenteeism, as well as being life threatening during attacks.^(^ 6 ^-^ 9 ^)^

Although there have been effective medications for the treatment of asthma since the 1980s, complete symptom control is not achieved in most patients.^(^ 10 ^,^ 11 ^)^ Because of their anti-inflammatory activity, inhaled corticosteroids are the therapy of choice in asthma. Maintenance treatment with an inhaled corticosteroid reduces the frequency and severity of exacerbations and the number of hospitalizations and emergency room visits, as well as improving quality of life, pulmonary function, and bronchial hyperresponsiveness and decreasing exercise-induced bronchoconstriction.^(^ 2 ^)^ Despite all the known benefits of this treatment, the Asthma Insights and Reality in Latin America (AIRLA) survey reported that only 6% of the asthma patients were using an inhaled corticosteroid.^(^ 12 ^)^ Inadequate maintenance treatment has a direct influence on the rate of disease control. Many studies have shown that, even in the 2000s, only one third of the asthma patients had totally controlled asthma.^(^ 13 ^,^ 14 ^)^ In the AIRLA survey, only 2.6% of the patients had well-controlled asthma.^(^ 12 ^)^

Several guidelines have been published to disseminate the appropriate management of asthma, on the basis of current clinical evidence.^(^ 2 ^,^ 8 ^)^ They contain objective strategies for assessing and measuring asthma control, as well as therapeutic recommendations and plans for asthma-related education. Those guidelines are expected to make it possible to reduce the impact of asthma on the lives of patients and to achieve complete asthma control.

In 2011, the Latin America Asthma Insight and Management (LA AIM) survey^(^ 15 ^)^ was designed to assess the impact of asthma on the lives of patients, their perception of their symptoms, and the prescribed treatment for the disease. The results of that survey made it possible to assess asthma control in those patients in accordance with the Global Initiative for Asthma (GINA) guidelines.^(^ 8 ^)^

The present study analyzed the data collected by the LA AIM survey in Brazil in order to determine the medications used (maintenance and rescue medications) and compliance with treatment, as well as to relate these variables to the level of asthma control.

## Methods

In 2011, the LA AIM survey, conducted in five Latin American countries (Argentina, Brazil, Mexico, Venezuela, and Puerto Rico), was designed using the same methods as the Asthma Insight and Management Study (AIM) conducted in the United States.^(^ 16 ^)^ The present study is based on the analysis of the Brazilian data from the LA AIM survey.

In Brazil, 4,545 households were randomly selected from a national probability sample in four cities: São Paulo; Rio de Janeiro; Curitiba; and Salvador. The population surveyed consisted of adults over 18 years of age and parents/caregivers of adolescents between 12 and 17 years of age; the individuals selected should have physician-diagnosed asthma. After the households that were initially selected were contacted by phone, 400 asthma patients were selected to be interviewed in person. The interviews lasted a maximum of 35 min. The questionnaire consisted of 53 questions that addressed five asthma topics: symptoms; impact of asthma on daily activities; perception of asthma control; exacerbations; and treatment and medications.

The questions about symptoms covered daytime and nighttime symptom frequency in the past four weeks, symptom frequency during the worst month of the past 12 months, the most bothersome symptom, triggering symptoms, symptom seasonality, and the frequency of symptom worsening. Respondents were asked whether they or their children had sought medical attention during exacerbations, symptom worsening, or severe acute episodes in the past year.

Regarding treatment, respondents were asked about the use of maintenance and rescue medications in the past four weeks and whether their physician had provided a written action plan for asthma management. Patients were investigated as to whether they used maintenance medication every day, whether that medication was necessary when the symptoms were not present, and whether rescue medication could be used every day, if necessary.

The present study was approved by the Research Ethics Committee of the Federal University of São Paulo *Hospital São Paulo* (Ruling no. 250155).

### Statistical analysis

Categorical data are presented as absolute values and percentages, and continuous data are presented as mean and standard deviation. The chi square test was used for the comparison of categorical data among the groups studied (controlled, partially controlled, and uncontrolled asthma), and ANOVA was used for the comparison of means. Tukey's post hoc test was used. A value of p < 0.05 was considered statistically significant.

## Results

The characteristics of the asthma patients in Brazil are shown in Table 1, by groups determined by GINA classification. In that Brazilian sample, the proportion of asthma patients was 8.8%. In accordance with the GINA criteria for asthma control, asthma was classified as controlled in 37 patients (9.3%), as partially controlled in 226 (56.5%), and as uncontrolled in 137 (34.3%).

**Table 1:** Demographic and epidemiological data of the respondents.a

Variables	Groups			p
	CA	PCA	UA	
	(n = 37)	(n = 226)	(n = 137)	
Age, years^b^	31.1 ± 9.9	38.5 ± 16.4	39.3 ± 16.6	0.03*
Female gender	24 (64.9)	143 (63.3)	105 (76.6)	0.02
Pets	20 (54.1)	115 (51.3)	64 (46.7)	0.59
Smoking status				
Smoker	7 (18.9)	60 (26.5)	29 (21.2)	0.007
Former smoker	2 (5.4)	56 (24.8)	45 (32.8)	
Never smoker	28 (75.7)	110 (48.7)	62 (45.3)	
Smoker(s) in the household	14 (37.8)	101 (44.7)	57 (41.6)	0.67

CA: controlled asthma; PCA: partially controlled asthma; and UA: uncontrolled asthma. aValues expressed as n (%), except where otherwise indicated. bValues expressed as mean ± SD. *CA group vs. UA group

The mean age was lower in the controlled asthma group than in the uncontrolled asthma group (31.1 ± 9.9 years vs. 39.3 ± 16.6 years; p = 0.03). In all groups, females predominated, especially in the uncontrolled asthma group (76.6%; p = 0.02). Most patients had pets, regardless of the group. In the controlled asthma group, 75.6% of the patients were never smokers. The presence or absence of smokers in the household did not affect asthma control (Table 1).

The medications used in the treatment of asthma are shown in Table 2. Regarding the use of maintenance medication in the past four weeks, 94.6% of the patients with controlled asthma reported that they were not using any maintenance medication regularly, whereas 80.1% of those with partially controlled asthma and 58.4% of those with uncontrolled asthma stated the same (p < 0.001). The use of rescue medication (short-acting β_2_ agonists) progressively increased as a function of poor asthma control, with 86.9% of the patients with uncontrolled asthma reporting their use (p < 0.001).

**Table 2:** Medications used in the treatment of asthma by the patients in the groups studied.a

Variables	Groups			p
	CA	PCA	UA	
	(n = 37)	(n = 226)	(n = 137)	
Maintenance medication in the past 4 weeks	2 (5.4)	45 (19.9)	57 (41.6)	< 0.001
Rescue medication in the past 4 weeks	5 (13.5)	98 (43.4)	119 (86.9)	< 0.001
Oral corticosteroid in the past 12 months during an asthma attack	17 (45.9)	92 (40.7)	77 (56.2)	0.06

CA: controlled asthma; PCA: partially controlled asthma; and UA: uncontrolled asthma. aValues expressed as n (%)

Oral corticosteroid use during an asthma attack was assessed in the past 12 months. The rates of oral corticosteroid use during an asthma attack were 45.9%, 40.7%, and 56.2%, respectively, in the controlled, partially controlled, and uncontrolled asthma groups, without significant difference (p = 0.06).

During the personal interview, participants reported what medications they had used for maintenance treatment of asthma and relief of symptoms in the past four weeks. Subsequently, a list of trade names of medications was presented to participants so that they could indicate the one(s) that they were using for maintenance treatment. The exact same list was presented to patients so that they could then identify the medication(s) used for relief of symptoms.

Most patients in the controlled asthma group reported that they were not using maintenance medication (55.6%), whereas among those in the partially controlled and uncontrolled asthma groups, the most commonly used medication was a short-acting bronchodilator (35.8% and 53.3%, respectively). Only 2.8% of the individuals in the controlled asthma group were using an inhaled corticosteroid, either alone or in combination with a long-acting β_2_ agonist. The rate of inhaled corticosteroid use was also very low in the partially controlled and uncontrolled asthma groups (12.9% and 24.1%, respectively). The use of a long-acting β_2_ agonist alone was reported by 5.6% and 3.5% of the patients in the controlled and partially controlled asthma groups, respectively. Only one patient in the partially controlled asthma group reported using tiotropium (Table 3).

**Table 3:** Maintenance mediations used for asthma control in the past four weeks according to the patients or their parents/guardians.a

Variables	Groups		
	CA	PCA	UA
	(n = 36)	(n = 226)	(n = 137)
None	20 (55.6)	81 (35.8)	20 (14.6)
Short-acting bronchodilator	12 (33.3)	81 (35.8)	73 (53.3)
Tiotropium	0 (0.0)	1 (0.4)	0 (0.0)
IC alone	1 (2.8)	11 (4.9)	12 (8.8)
LAB + IC	0 (0.0)	18 (8.0)	21 (15.3)
LAB alone	2 (5.6)	8 (3.5)	0 (0.0)
Aminophylline	0 (0.0)	6 (2.7)	4 (2.9)
Unknown	0 (0.0)	11 (4.9)	3 (2.2)
Others	1 (2.8)	9 (4.0)	4 (2.9)

CA: controlled asthma; PCA: partially controlled asthma; UA: uncontrolled asthma; IC: inhaled corticosteroid; and LAB: long-acting bronchodilator. aValues expressed as n (%)

Regarding rescue medications, 12.1% and 10.9% of the individuals in the controlled and partially controlled asthma groups, respectively, reported that they were not using any rescue medication. In contrast, all patients in the uncontrolled asthma group used at least one medication they referred to as rescue medication. The vast majority of patients in the three groups used a short-acting bronchodilator (66.7%, 71.5%, and 80.6% in the controlled, partially controlled, and uncontrolled asthma groups, respectively). An inhaled corticosteroid was considered rescue medication by some patients (3.0%, 2.3%, and 4.5% in the same groups, respectively), as were the combination of an inhaled corticosteroid and a long-acting β_2_ agonist (0.0%, 3.2%, and 9.0%, respectively) and a long-acting β_2_ agonist alone (9.1%, 2.7%, and 0.0%, respectively; Table 4).

**Table 4:** Rescue medications used for asthma control in the past four weeks according to the patients or their parents/guardians.a

Variables	Groups		
	CA	PCA	UA
	(n = 33)	(n = 221)	(n = 134)
None	4 (12.1)	24 (10.9)	0 (0.0)
Short-acting bronchodilator	22 (66.7)	158 (71.5)	108 (80.6)
IC alone	1 (3.0)	5 (2.3)	6 (4.5)
LAB + IC	0 (0.0)	7 (3.2)	12 (9.0)
LAB alone	3 (9.1)	6 (2.7)	0 (0.0)
Aminophylline	2 (6.1)	8 (3.6)	3 (2.2)
Unknown	0 (0.0)	8 (3.6)	1 (0.7)
Others	1 (3.0)	5 (2.3)	4 (3.0)

CA: controlled asthma; PCA: partially controlled asthma; UA: uncontrolled asthma; IC: inhaled corticosteroid; and LAB: long-acting bronchodilator. aValues expressed as n (%).

Those patients who had asthma attacks in the past 12 months were asked whether, during an attack, their use of rescue medication was higher than, lower than, or equal to their daily use. All groups reported an increased need to use rescue medication (30.0%, 64.9%, and 57.1% of the patients in the controlled, partially controlled, and uncontrolled asthma groups, respectively), although the increase was more pronounced in the partially controlled and uncontrolled asthma groups (p = 0.002 in relation to the controlled asthma group; data not shown in tables).

In the sample as a whole, 42.0% of the asthma patients reported that their physician had provided a written treatment action plan, explaining the need for maintenance treatment and when to use the rescue medication.

In addition, 41% of the patients reported that they fully or partially agreed with the idea that the rescue medication should be used daily, regardless of the presence of symptoms. Regarding continuous inhaled corticosteroid use, 62.3% of the patients said that it was a cause for concern, and, when categorized into groups, 51.3%, 57.5%, and 73.0% of the patients in the controlled, partially controlled, and uncontrolled asthma groups, respectively, reported that concern. The reasons given by patients for their fear of using an inhaled corticosteroid were its side effects, concern about its safety and long-term effects, and concern about dependence.

## Discussion

The present study has shown that, in accordance with GINA criteria,^(^ 8 ^)^ asthma is uncontrolled in the vast majority of asthma patients in Brazil. A small proportion of patients in the uncontrolled and partially controlled asthma groups used maintenance medication, and, consequently, they were the ones who used oral corticosteroids and rescue medication the most often in the past 12 months.

Because the inclusion criterion for the present study was having received a physician diagnosis of asthma, the observed proportion of asthma patients was 8.8%, which is very similar to the prevalence of physician-diagnosed asthma in Brazil, according to the International Study of Asthma and Allergies in Childhood.^(^ 17 ^)^ Therefore, it is possible that our results reflect the national situation regarding asthma control. Nevertheless, the objective of the present study was not to assess the prevalence of asthma in Brazil, but rather to assess patient's use of medications.

The poor control of asthma in Latin America has been known since 2003, when the AIRLA survey showed that asthma was totally controlled in only 2.6% of the adult patients with asthma and in 2.4% of the children with asthma.^(^ 12 ^)^ The design of our study was similar to that of the AIRLA survey, and, although our study used other criteria for classifying asthma control (daytime and nighttime symptoms, exercise-induced symptoms, and overall severity of symptoms), the two studies showed similar results. The fact that the current number of patients with controlled asthma is approximately nearly three times that of 10 years ago, according to the AIRLA survey, should not be seen as encouraging, because, for a disease for which treatment and treatment effectiveness are well known, 8% is a very low number.

Despite widespread knowledge that inhaled steroids are the medication of choice as asthma controllers, we found that most patients did not use them. The fact that only 5.4% of the patients with controlled asthma had used maintenance medication in the past four weeks may reflect that this group consists of patients with mild disease, not requiring continuous inhaled corticosteroid use. However, approximately half of those patients had to take an oral corticosteroid during an asthma attack, which demonstrates that, at some time in the year, asthma was uncontrolled in those patients. Only 19.9% of the patients with partially controlled asthma and 41.6% of those with uncontrolled asthma reported that they had used maintenance medication in the past month, despite the fact that they reported rescue medication use. In the AIM survey conducted in the United States in 2009,^(^ 18 ^)^ the observed proportion of patients who used maintenance medication in the controlled asthma group in that country (32%) was higher than that in Brazil. The fact that inhaled corticosteroids were used by a higher proportion of American patients than of Brazilian patients could be the reason for the lower rate of hospital visits and admissions among American patients with asthma. Likewise, the proportion of patients who used rescue medication in the United States was lower in all groups, which reinforces the idea that asthma is better controlled in patients who use maintenance medication more often.^(^ 18 ^)^ A study conducted at a referral center for the treatment of pediatric asthma in Brazil reported that symptoms were controlled in 45% of the patients.^(^ 19 ^)^ This shows us that, even at a referral center for the treatment of asthma, although the reported rate of disease control was much higher than that found in the present study, asthma control was achieved in only half of the patients.

In 2003, the rate of inhaled corticosteroid use in Latin America was 6%.^(^ 12 ^)^ In the present study, 15.8% of the patients had been on maintenance therapy in the past four weeks, when assessed by medication use. Although this rate is below ideal, with the ideal scenario being inhaled corticosteroid use by all patients with partially controlled or uncontrolled asthma, it is two and a half times higher than that reported by the AIRLA survey, demonstrating a significant improvement towards meeting the goal established in the guidelines. The low number of patients who used an inhaled corticosteroid could reflect two situations: physicians are not prescribing controller medication properly, which is in clear noncompliance with the Brazilian Thoracic Association guidelines^(^ 2 ^)^ and the GINA guidelines;^(^ 8 ^)^ or patients are poorly compliant with maintenance therapy, which would demonstrate their poor understanding of the disease and its treatment. When asked whether they had some concern about using an inhaled corticosteroid continuously, 62.3% of the patients responded affirmatively, which shows us that most patients were not informed of or did not understand the importance of maintenance treatment of asthma. This demonstrates that greater educational intervention is needed to resolve patient uncertainties about the safety of the medication, its possible side effects, and its long-term benefits. Other factors that may explain the poor treatment compliance are poor symptom perception by the patients, difficult access to medical appointments, medication cost, and medication dose schedule.

A study conducted at a referral center for the treatment of severe asthma in the state of Bahia,^(^ 20 ^)^ Brazil, in which educational strategies were used, reported high compliance with inhaled corticosteroid use (83.8%). This shows that the use of appropriate patient education strategies makes it possible to achieve optimal treatment. The factors related to patient noncompliance in that study were medication adverse effects, living far from the referral center, limited resources to pay for transportation to and from the center, and dose schedule.^(^ 20 ^)^

Analysis of Tables 3 and 4, which depict the medications reported by patients as being used, shows that there is clear confusion between maintenance and rescue therapy, i.e., patients have difficulty acknowledging the role of each type of therapy in the treatment of asthma. Some patients were using an inhaled corticosteroid alone for relief of symptoms. A cause for even greater concern is that there were patients (5.6% and 3.5% in the controlled and partially controlled asthma groups, respectively) who were using a long-acting β_2_ agonist alone, which is absolutely advised against in the treatment of asthma. Once again, the lack of understanding of the disease and its treatment on the part of patients is evident.

Rescue short-acting bronchodilator use is one of the markers of asthma severity, and, in the present study, the patients in greatest need for rescue medication use were found to be those in the partially controlled and uncontrolled asthma groups. An assessment of the medications used for relief of symptoms in the past four weeks, as indicated by the name of the medication, showed that their rate of use, in the three groups, ranged from 66% to 80% (Table 4). This is another indication of underuse of maintenance medication, because it reflects the high number of patients who used rescue medication. This high rate of rescue medication use shows that patients were still having asthma attacks, suggesting that the doses of maintenance medication were insufficient. One limitation of the administered questionnaire in terms of short-acting bronchodilator use was that patients were asked solely whether they had or had not used the medication in the past four weeks, rather than being asked about the frequency of use. This information was partially taken into account when classifying asthma control.

Oral corticosteroid use, another marker of poor asthma control, shows a trend toward being increased in the partially controlled and uncontrolled asthma groups. The need for oral corticosteroid use, in all groups, was higher in Brazil than in the United States (controlled asthma, 45.9% vs. 15%; partially controlled asthma, 40.7% vs. 40%; uncontrolled asthma, 56.2% vs. 45%). ^(^ 18 ^) ^In 2009, a study of asthma education that was conducted in Brazil and followed patients over a two-year period reported an association between reduced oral corticosteroid use and increased compliance with maintenance treatment, emphasizing the importance of educational interventions for asthma control.^(^ 21 ^)^

It is of interest that the number of patients who reported having a written treatment plan in Brazil (41%) was higher than that in the United States (32%).^(^ 15 ^)^ In the AIRLA survey,^(^ 12 ^)^ in 2003, the observed proportion of adult patients who had a prepared action plan (38%) was very similar to the current number, which demonstrates that, despite the Brazilian Thoracic Association guidelines^(^ 2 ^)^ and the GINA guidelines,^(^ 8 ^)^ there has been no progress on this issue.

The design of the present study was the same as that of the AIM survey^(^ 16 ^)^ and was similar to that of the AIRLA survey.^(^ 12 ^)^ However, some limitations were found in our study. Only four Brazilian cities were assessed in the study, which means that the study sample may not be representative of the general population in Brazil. It is very difficult to cover the entire population of a country in studies with this type of design, though. Nevertheless, because each of the included cities is located in a different area of the country, this limitation is mitigated. Another limitation was the fact that spirometry, a test that is also part of the criteria for asthma control, was not performed. However, given that the primary objective of the study was to characterize the reality of patients and their daily routine, classification of asthma control solely on the basis of the questionnaire responses was enough to show that the goal of attaining disease control has yet to be achieved in asthma management. Finally, the data on the diagnosis of asthma and the other data were obtained by self-report rather than from medical record abstraction.

On the basis of this study, we conclude that, in accordance with GINA criteria, asthma is not adequately controlled in the vast majority of asthma patients in Brazil, and this poor control should be attributed to underuse of maintenance medication. Consequently, we found that rescue medications and oral corticosteroids are very often used by patients with partially controlled or uncontrolled asthma. Therefore, greater efforts should be made to ensure that proper asthma treatment is prescribed and that compliance with the treatment plan is achieved.
