# Supplementary material for: Level of asthma control and its relationship with medication use in asthma patients in Brazil
Source: J Bras Pneumol. 2014 Sep-Oct;40(5):487–94. doi: 10.1590/S1806-37132014000500004 (PMC4263329; doi:10.1590/S1806-37132014000500004)
Supplement: Translation

# Translation: Nível de controle da asma e sua relação com o uso de medicação em asmáticos no Brasil*

**Authors:** Josiane Marchioro, Mariana Rodrigues Gazzotti, Oliver Augusto Nascimento, Federico Montealegre, James Fish, José Roberto Jardim

**Affiliations:** Escola Paulista de Medicina, Universidade Federal de São Paulo, São Paulo (SP) Brasil; Escola Paulista de Medicina, Universidade Federal de São Paulo, São Paulo (SP) Brasil; Escola Paulista de Medicina, Universidade Federal de São Paulo, São Paulo (SP) Brasil; Merck, Sharp & Dohme Corp., São Paulo (SP) Brasil; Merck, Sharp & Dohme Corp., São Paulo (SP) Brasil; Escola Paulista de Medicina, Universidade Federal de São Paulo, São Paulo (SP) Brasil

**Keywords:** Asma/terapia, Asma/prevenção e controle, Adesão à medicação

## Abstract

**OBJETIVO:**

Avaliar pacientes asmáticos no Brasil em relação ao grau de controle da asma, a aderência ao tratamento de manutenção e o uso de medicação de alivio em pacientes asmáticos.

**MÉTODOS:**

Foram utilizados os dados de um inquérito latino-americano, obtidos em quatro capitais brasileiras, de 400 pacientes com asma através de um questionário sobre o controle e o tratamento da doença.

**RESULTADOS:**

A prevalência de asma nesta amostra foi de 8,8%. Dos 400 pacientes estudados, 37 (9,3%), 226 (56,5%) e 137 (34,3%), respectivamente, foram classificados, segundo critérios da *Global Initiative for Asthma*, como tendo asma controlada, parcialmente controlada e não controlada. A proporção de pacientes em terapia de manutenção nas últimas quatro semanas naqueles três grupos, respectivamente, foi de 5,4%, 19,9% e 41,6%. O uso de medicação de alivio foi significativamente mais comum nos pacientes com asma não controlada (86,9%; p < 0,001).

**CONCLUSÕES:**

Nossos achados sugerem que a grande maioria dos pacientes com asma no Brasil não apresenta sua doença controlada segundo critérios internacionais. As medicações de manutenção ainda são subutilizadas no Brasil, e o uso de medicações de alívio e corticoide oral é mais frequente em pacientes com asma parcialmente controlada ou não controlada.

## Introdução

A asma é uma doença crônica comum em todo o mundo, que acomete indivíduos de todas as faixas etárias e tem grande influência na qualidade de vida dos pacientes.^(^ 1 ^,^ 2 ^)^ Em 2006, o *International Study of Asthma and Allergies in Childhood* mostrou uma prevalência de asma de 24,3% em escolares e 19,0% em adolescentes no Brasil,^(^ 3 ^,^ 4 ^)^ ao passo que a prevalência de asma diagnosticada por médico foi de aproximadamente 10%. A asma, por sua alta prevalência, tem um grande impacto socioeconômico,^(^ 5 ^)^ uma vez que, quando não controlada, pode gerar hospitalizações, abstenção à escola e/ou ao trabalho, além de acarretar risco de vida durante as crises.^(^ 6 ^-^ 9 ^)^

Apesar de existirem medicações efetivas para o tratamento da asma desde os anos 80, o controle completo dos sintomas não é obtido na maioria dos pacientes.^(^ 10 ^,^ 11 ^)^ O corticoide inalatório, por sua ação anti-inflamatória, é a terapia de eleição na asma. O tratamento de manutenção com corticoide inalatório reduz a frequência e a gravidade das exacerbações, o número de hospitalizações e de atendimentos nos serviços de emergência, assim como melhora a qualidade de vida, a função pulmonar e a hiper-responsividade brônquica e diminui a broncoconstrição induzida pelo exercício.^(^ 2 ^)^ Mesmo conhecendo-se todos os benefícios desse tratamento, o estudo *Asthma Insights and Reality in Latin America* (AIRLA) mostrou que apenas 6% dos pacientes asmáticos estavam em uso de corticoide inalatório.^(^ 12 ^)^ O tratamento de manutenção inadequado influi diretamente na taxa de controle da doença. Muitos estudos mostraram que, mesmo nos anos 2000, apenas um terço dos asmáticos tem sua doença totalmente controlada.^(^ 13 ^,^ 14 ^)^ No estudo AIRLA, somente 2,6% dos pacientes tinham asma bem controlada.^(^ 12 ^)^

Várias diretrizes têm sido publicadas com a finalidade de divulgar o manejo adequado da doença, segundo as atuais evidências clínicas,^(^ 2 ^,^ 8 ^)^ contendo estratégias objetivas para avaliar e mensurar o controle da doença, as recomendações terapêuticas e os planos para educação relacionada à asma. A expectativa, com essas diretrizes, é reduzir o impacto da asma na vida dos pacientes e atingir o controle completo da doença. 

Em 2011, o inquérito *Latin America Asthma Insight and Management* (LA AIM) foi delineado para avaliar o impacto da asma na vida dos pacientes, a sua percepção em relação aos seus sintomas e a terapia prescrita para a doença.^(^ 15 ^)^ Com os resultados desse inquérito foi possível avaliar o controle da asma daqueles pacientes segundo as diretrizes da *Global Initiative for Asthma* (GINA).^(^ 8 ^)^

O presente estudo analisou os dados colhidos pelo LA AIM no Brasil e teve como objetivo avaliar as medicações utilizadas, tanto de manutenção quanto de alivio, e a aderência ao tratamento, assim como relacionar essas variáveis ao grau de controle da doença.

## Métodos

Em 2011, o inquérito LA AIM, realizado em cinco locais na América Latina (Argentina, Brasil, México, Venezuela e Porto Rico) foi delineado seguindo os mesmos métodos empregados no *Asthma Insight and Management Study* (AIM) realizado nos EUA.^(^ 16 ^)^ O presente estudo baseia-se na análise dos dados brasileiros do estudo AIM latino-americano.

Foram selecionados no Brasil, aleatoriamente, 4.545 domicílios, utilizando-se uma amostragem probabilística nacional, em quatro cidades: São Paulo, Rio de Janeiro, Curitiba e Salvador. A população utilizada foi composta por adultos acima de 18 anos e pais/cuidadores de adolescentes entre 12 e 17 anos; os indivíduos selecionados deveriam ter asma diagnosticada por um médico. Das residências selecionadas inicialmente, após um contato telefônico, selecionaram-se 400 asmáticos para serem entrevistados pessoalmente. As entrevistas tiveram duração máxima de 35 min. O questionário continha 53 questões, abordando cinco tópicos sobre asma: sintomas; impacto da doença nas atividades diárias; percepção do controle da asma; exacerbações; e tratamento e medicações.

As questões sobre os sintomas avaliaram a frequência de sintomas diurnos e noturnos nas últimas quatro semanas, a frequência de sintomas durante o pior mês dos últimos 12 meses, o sintoma mais incômodo, os sintomas gatilho, se os sintomas eram ou não sazonais e a frequência de agravamento dos sintomas. Os participantes eram questionados se eles ou seus filhos consultaram um médico durante as exacerbações, piora dos sintomas ou episódios agudos graves no último ano.

A respeito do tratamento, perguntou-se sobre o uso de medicações de manutenção e de alívio nas últimas quatro semanas e se o médico forneceu um plano de ação para o manejo da doença por escrito. Investigou-se se o paciente usava a medicação de manutenção todos os dias, se essas eram necessárias quando os sintomas não estavam presentes e se a medicação de alívio poderia ser utilizada todos os dias, se necessário.

O presente estudo foi aprovado pelo Comitê de Ética em Pesquisa do Hospital São Paulo/Universidade Federal de São Paulo sob o parecer no. 250155.

### Análise estatística

Os dados categóricos são apresentados em valor absoluto e proporção, e os dados numéricos, em média e desvio-padrão. Para a comparação de dados categóricos entre os grupos (pacientes com asma controlada, parcialmente controlada e não controlada) foi utilizado o teste do qui-quadrado, e para comparação das médias foi utilizada ANOVA. O teste *post hoc* adotado foi o de Tukey. Foi considerado estatisticamente significante p < 0,05.

## Resultados

As características dos pacientes com asma no Brasil, de acordo com a classificação de GINA, são mostradas na Tabela 1. A proporção de asmáticos na presente amostra brasileira foi de 8,8%. De acordo com os critérios de controle da asma por GINA, a asma foi classificada como controlada, em 37 pacientes (9,3%); em parcialmente controlada, em 226 (56,5%); e não controlada, em 137 (34,3%).

**Tabela 1:** Dados demográficos e epidemiológicos dos entrevistados.a

Variáveis	Grupos			p
	AC	APC	ANC	
	(n = 37)	(n = 226)	(n = 137)	
Idade, anos^b^	31,1 ± 9,9	38,5 ± 16,4	39,3 ± 16,6	0,03*
Gênero feminino	24 (64,9)	143 (63,3)	105 (76,6)	0,02
Animais domésticos	20 (54,1)	115 (51,3)	64 (46,7)	0,59
Estado tabágico				
Fumante	7 (18,9)	60 (26,5)	29 (21,2)	0,007
Ex-fumante	2 (5,4)	56 (24,8)	45 (32,8)	
Nunca fumaram	28 (75,7)	110 (48,7)	62 (45,3)	
Fumante no domicílio	14 (37,8)	101 (44,7)	57 (41,6)	0,67

: asma controlada; APC: asma parcialmente controlada; e ANC: asma não controlada

a Valores expressos em n (%), exceto onde indicado

b Valores expressos em média ± dp

* Grupo AC vs. grupo ANC.

Os pacientes com asma controlada tiveram média de idade inferior à dos pacientes com asma não controlada (31,1 ± 9,9 anos vs. 39,3 ± 16,6 anos; p = 0,03). Em todos os grupos houve predomínio do sexo feminino, principalmente no grupo asma não controlada (76,6%; p = 0,02). A maioria dos pacientes possuía animais domésticos, independentemente dos grupos de controle da doença. Em relação ao status tabágico, 75,6% dos pacientes com asma controlada nunca fumaram. A presença ou não de fumantes no domicílio não influenciou no controle da asma (Tabela 1).

As medicações utilizadas para o tratamento da asma são mostradas na Tabela 2. Em relação ao uso de medicação de manutenção nas últimas quatro semanas, 94,6% dos pacientes com doença controlada afirmaram não estar em uso regular de nenhuma medicação de manutenção, enquanto 80,1% dos pacientes parcialmente controlados e 58,4% dos pacientes não controlados fizeram a mesma afirmação (p < 0,001). Quanto ao uso de medicação de alívio (β_2_-agonista de curta duração), o seu uso foi progressivamente aumentando em função da falta de controle da asma dos pacientes, chegando a 86,9% nos pacientes com asma não controlada (p < 0,001).

**Tabela 2:** Medicações utilizadas no tratamento da asma pelos pacientes nos grupos estudados.a

Variáveis	Grupos			p
	AC	APC	ANC	
	(n = 37)	(n = 226)	(n = 137)	
Medicação de manutenção nas últimas 4 semanas	2 (5,4)	45 (19,9)	57 (41,6)	< 0,001
Medicação de alívio nas últimas 4 semanas	5 (13,5)	98 (43,4)	119 (86,9)	< 0,001
Corticoide oral nos últimos 12 meses durante crise de asma	17(45,9)	92 (40,7)	77 (56,2)	0,06

AC: asma controlada; APC: asma parcialmente controlada; e ANC: asma não controlada

a Valores expressos em n (%).

O uso de corticoide oral durante as crises foi avaliado nos últimos 12 meses. As taxas de uso de corticoide oral durante as crises foram de 45,9%, 40,7% e 56,2% nos grupos asma controlada, parcialmente controlada e não controlada, respectivamente, sem diferença significativa (p = 0,06).

Durante a entrevista pessoal, os participantes informaram quais as medicações que eles estavam utilizando nas últimas quatro semanas para a manutenção e para o alívio dos sintomas da asma. Em seguida, apresentou-se uma lista com os nomes comerciais das medicações para que o participante indicasse qual(is) estava(m) em uso para a manutenção da doença. Exatamente a mesma lista era apresentada ao paciente para que, então, identificasse a(s) medicação(ões) usada(s) para o alívio dos sintomas.

A maioria dos pacientes com asma controlada afirmou não estar em uso de medicação de manutenção (55,6%), enquanto nos grupos asma parcialmente controlada e não controlada, a medicação mais utilizada foi um broncodilatador de curta duração (35,8% e 53,3%, respectivamente). Apenas 2,8% dos indivíduos do grupo asma controlada estavam em uso de corticoide inalatório, seja de forma isolada, seja associado a um β_2_-agonista de ação prolongada. A taxa de uso de corticoide inalatório também foi muito baixa nos grupos de asma parcialmente controlada e não controlada (12,9% e 24,1%, respectivamente). Em relação ao uso de β_2_-agonista de ação prolongada isoladamente, 5,6% e 3,5% dos pacientes nos grupos asma controlada e parcialmente controlada, respectivamente, faziam seu uso. Apenas um paciente do grupo asma parcialmente controlada informou estar em uso de tiotrópio (Tabela 3).

**Tabela 3:** Medicações de manutenção utilizadas nas últimas quatro semanas para o controle da asma segundo os pacientes ou seus pais/responsáveis.a

Variáveis	Grupos		
	AC	APC	ANC
	(n = 36)	(n = 226)	(n = 137)
Nenhuma	20 (55,6)	81 (35,8)	20 (14,6)
Broncodilatador de curta duração	12 (33,3)	81 (35,8)	73 (53,3)
Tiotrópio	0 (0,0)	1 (0,4)	0 (0,0)
CI isolado	1 (2,8)	11 (4,9)	12 (8,8)
BLD + CI	0 (0,0)	18 (8,0)	21 (15,3)
BLD isolado	2 (5,6)	8 (3,5)	0 (0,0)
Aminofilina	0 (0,0)	6 (2,7)	4 (2,9)
Não soube informar	0 (0,0)	11 (4,9)	3 (2,2)
Outros	1 (2,8)	9 (4,0)	4 (2,9)

AC: asma controlada; APC: asma parcialmente controlada; ANC: asma não controlada; CI: corticoide inalatório; BLD: broncodilatador de longa duração

a Valores expressos em n (%).

Quanto às medicações de alívio, 12,1% e 10,9% dos indivíduos nos grupos asma controlada e parcialmente controlada, respectivamente, afirmaram não estar em uso de nenhuma medicação de alívio. Já no grupo asma não controlada, todos os pacientes utilizavam pelo menos uma medicação referida como de alívio. A grande maioria dos pacientes fazia uso de broncodilatador de curta duração nos três grupos (66,7%, 71,5% e 80,6% nos grupos asma controlada, parcialmente controlada e não controlada, respectivamente). Alguns pacientes consideravam como medicações de alívio o corticoide inalatório (3,0%, 2,3% e 4,5% nos mesmos grupos, respectivamente), a combinação corticoide inalatório + β_2_-agonista de ação prolongada (0,0%, 3,2% e 9,0%, respectivamente) e β_2_-agonista de ação prolongada isoladamente (em 9,1%, 2,7% e 0,0%, respectivamente; Tabela 4).

**Tabela 4:** Medicações de alívio utilizadas nas últimas quatro semanas para o controle da asma segundo os pacientes ou seus pais/responsáveis.a

Variáveis	Grupos		
	AC	APC	ANC
	(n = 33)	(n = 221)	(n = 134)
Nenhuma	4 (12,1)	24 (10,9)	0 (0,0)
Broncodilatador de curta duração	22 (66,7)	158 (71,5)	108 (80,6)
CI isolado	1 (3,0)	5 (2,3)	6 (4,5)
BLD + CI	0 (0,0)	7 (3,2)	12 (9,0)
BLD isolado	3 (9,1)	6 (2,7)	0 (0,0)
Aminofilina	2 (6,1)	8 (3,6)	3 (2,2)
Não soube informar	0 (0,0)	8 (3,6)	1 (0,7)
Outros	1 (3,0)	5 (2,3)	4 (3,0)

AC: asma controlada; APC: asma parcialmente controlada; ANC: asma não controlada; CI: corticoide inalatório; BLD: broncodilatador de longa duração

a Valores expressos em n (%).

Para aqueles pacientes que tiveram crises de asma nos últimos 12 meses, perguntou-se se, durante a crise, o uso de medicação de resgate era maior, menor ou igual ao usado no seu dia a dia. Todos os grupos referiram um aumento na demanda de uso da medicação de alívio (em 30,0%, 64,9% e 57,1% dos pacientes nos grupos asma controlada, parcialmente controlada e não controlada, respectivamente), embora ele tenha sido mais acentuado nos grupos asma parcialmente controlada e não controlada (p = 0,002 em relação ao grupo asma controlada; dados não mostrados em tabela).

Da amostra geral, 42,0% dos pacientes com asma relataram que seu médico havia feito um plano de ação terapêutica por escrito, descrevendo a necessidade de uso de terapia de manutenção e quando utilizar a medicação de resgate. 

Além disso, 41% dos pacientes informaram concordar parcialmente ou totalmente com a ideia de que a medicação de manutenção deveria ser utilizada diariamente, independentemente da presença de sintomas. Em relação ao uso contínuo de corticoide inalatório, 62,3% dos pacientes disseram se preocupar com seu uso contínuo e, quando categorizados, essa preocupação foi referida por 51,3%, 57,5% e 73,0% dos pacientes nos grupos asma controlada, parcialmente controlada e não controlada, respectivamente. As razões para o medo de utilizar o corticoide inalatório apontadas pelos pacientes foram seus efeitos colaterais, a preocupação quanto a sua segurança e efeitos no longo prazo e a preocupação quanto à dependência.

## Discussão

O presente estudo mostra que a grande maioria dos pacientes asmáticos no Brasil não apresenta asma controlada, de acordo com os critérios de GINA.^(^ 8 ^)^ Uma pequena proporção dos pacientes dos grupos asma não controlada e parcialmente controlada utilizavam medicação de manutenção e, consequentemente, foram os que mais frequentemente usaram corticoide oral e medicação de alívio nos últimos 12 meses.

Como o critério de inclusão para o presente estudo era haver recebido o diagnóstico de asma por um médico, a proporção de asmáticos encontrada foi de 8,8%, muito semelhante à prevalência de asma diagnosticada por um médico no Brasil, de acordo com o *International Study of Asthma and Allergies in Childhood*.^(^ 17 ^)^ Portanto, é possível que os nossos resultados possam refletir o panorama nacional sobre o controle da asma. De qualquer modo, o objetivo do presente estudo não foi avaliar a prevalência de asma no Brasil mas o de avaliar a utilização de medicamentos pelos pacientes.

O mau controle da asma na América Latina já é conhecido desde 2003, quando o estudo AIRLA mostrou que apenas 2,6% dos pacientes adultos asmáticos e 2,4% das crianças asmáticas tinham a sua doença totalmente controlada.^(^ 12 ^)^ O nosso estudo teve um delineamento similar ao AIRLA e, apesar de usar outros critérios de classificação para o controle da asma (sintomas diurnos e noturnos, sintomas induzidos por exercício e gravidade total dos sintomas), os dois estudos apresentam resultados similares. O fato de haver, aproximadamente, quase três vezes mais asmáticos controlados atualmente do que há 10 anos, de acordo com estudo AIRLA, não deve ser visto como algo alentador, pois, para uma doença cujos tratamento e sua efetividade são bem conhecidos, 8% é um número muito baixo.

Apesar do amplo conhecimento de que os esteroides inalatórios são a medicação para o controle da asma, vimos que a maioria dos pacientes permaneceu sem o seu uso. O fato de apenas 5,4% dos pacientes com asma controlada estarem em uso de medicação de manutenção nas últimas quatro semanas deve refletir que esse grupo é constituído por pacientes com doença leve, não necessitando de uso contínuo de corticoide inalatório. No entanto, aproximadamente a metade desses pacientes teve de tomar corticoide oral durante alguma crise, o que demonstra que esses pacientes, em alguma época do ano, não estavam com a doença controlada. Apenas 19,9% dos pacientes com asma parcialmente controlada e 41,6% daqueles com asma não controlada relataram estar em uso de medicação de manutenção no último mês, a despeito de relatarem o uso de medicação de alívio. No estudo AIM realizado nos EUA em 2009,^(^ 18 ^)^ observou-se que o uso de medicação de manutenção no grupo de pacientes com asma controlada naquele país foi maior (32%) do que no Brasil. O uso de corticoide inalatório numa maior proporção de pacientes americanos do que de brasileiros talvez seja a causa da menor taxa de visitas hospitalares e internações dos asmáticos americanos. Do mesmo modo, o uso de medicação de alívio nos EUA foi menor em todos os grupos, o que reforça a ideia de que os pacientes que estão mais frequentemente em uso de medicação de manutenção estão mais bem controlados.^(^ 18 ^)^ Em um estudo realizado em um centro de referência para o tratamento da asma pediátrica no Brasil, relatou-se que 45% dos pacientes tinham seus sintomas controlados. ^(^ 19 ^)^ Isso nos mostra que, mesmo em um centro de referência para o tratamento da asma, apesar de a taxa de controle encontrada ter sido muito maior do que a identificada no presente estudo, o controle da doença só ocorreu em metade dos pacientes.

Em 2003, a taxa de uso de corticoide inalatório na América Latina era de 6%.^(^ 12 ^)^ No presente estudo, 15,8% dos pacientes estavam em terapia de manutenção nas últimas quatro semanas, quando avaliados pela medicação em uso. Apesar de essa taxa estar abaixo do ideal, que seria o uso do corticoide inalatório por todos os pacientes com asma parcialmente controlada ou não controlada, ela é duas vezes e meia mais alta do que a reportada pelo estudo AIRLA, demonstrando uma melhora significativa nessa meta estabelecida pelas diretrizes. O baixo número de pacientes em uso de corticoide inalatório pode refletir duas situações: os médicos não estão prescrevendo a medicação de controle adequadamente, o que está em flagrante desacordo com as diretrizes da Sociedade Brasileira de Pneumologia e Tisiologia^(^ 2 ^)^ e de GINA,^(^ 8 ^)^ e/ou os pacientes estão pouco aderentes à terapia de manutenção, o que demonstraria pouco entendimento sobre a doença e seu tratamento. Quando perguntados se tinham alguma preocupação em usar o corticoide inalatório continuamente, 62,3% dos pacientes responderam afirmativamente, o que nos mostra que a maioria dos pacientes não foi informada ou não compreendeu a importância do tratamento de manutenção da asma. Isso demonstra que é necessária uma maior intervenção educacional, visando esclarecer as dúvidas do paciente quanto à segurança da medicação, seus possíveis efeitos colaterais e seus benefícios em longo prazo. Outros fatores que podem explicar a baixa aderência ao tratamento são a baixa percepção dos sintomas pelo paciente, o difícil acesso às consultas médicas, o custo das medicações e sua posologia.

Um estudo conduzido em um centro de referência para o tratamento de asma grave no estado da Bahia^(^ 20 ^)^ em nosso país, no qual foram utilizadas estratégias educacionais, mostrou uma alta aderência do uso de corticoide inalatório (83,8%). Isso mostra que com o uso de estratégias adequadas de educação do paciente é possível alcançar o tratamento ideal. Os fatores relacionados aos pacientes não aderentes naquele estudo foram os efeitos adversos da medicação, o local de residência distante do centro de referência, a dificuldade de pagar pelo transporte até o centro e o regime posológico.^(^ 20 ^)^

Quando analisamos as Tabelas 3 e 4, que mostram as medicações em uso referidas diretamente pelos pacientes, vemos que há uma clara confusão entre terapia de manutenção e de alívio, ou seja, os pacientes têm dificuldades de reconhecer o papel de cada tipo de terapia no tratamento da asma. Alguns pacientes estavam utilizando corticoide inalatório de forma isolada para o alívio dos sintomas. Ainda mais preocupante, existem pacientes (5,6% e 3,5% dos grupos asma controlada e asma parcialmente controlada, respectivamente) que estavam em uso isolado de β_2_-agonista de ação prolongada, o que é absolutamente proscrito no tratamento da asma. Mais uma vez, fica evidente a falta de entendimento por parte dos pacientes sobre a doença e seu tratamento.

O uso de broncodilatador de curta duração de resgate é um dos marcadores de gravidade da asma e, no presente estudo, pode-se perceber que os pacientes com uma maior necessidade de uso da medicação de alívio eram aqueles dos grupos asma parcialmente controlada e asma não controlada. Quando avaliadas as medicações utilizadas para alívio nas últimas quatro semanas, de acordo com a indicação pelo nome da medicação, observamos que houve o uso dessa medicação, nos três grupos, que variou de 66% a 80% (Tabela 4). Isso é mais uma indicação do baixo uso de medicação de manutenção, porque reflete o elevado número de pacientes que utilizaram medicação de alívio. Essa alta taxa de uso de medicação de alívio mostra que eles ainda estavam tendo crises, levando a crer que a dose da medicação de manutenção poderia ser insuficiente. Uma limitação do questionário utilizado em relação ao uso de broncodilatador de curta duração foi que o paciente era questionado apenas se ele utilizou ou não a medicação nas últimas quatro semanas e não a frequência do uso. Esse dado foi levado em consideração de modo parcial para classificar o controle da asma.

Outro marcador de baixo controle da doença, o uso de corticoide oral, mostra a tendência ao seu maior uso nos grupos com doença parcialmente controlada e não controlada. A necessidade de uso de corticoide oral, em todos os grupos, foi maior no Brasil do que nos EUA (asma controlada, 45,9% vs. 15%; asma parcialmente controlada, 40,7% vs. 40%; asma não controlado, 56,2% vs. 45%).^(^ 18 ^)^ Em 2009, um estudo brasileiro sobre a educação em asma que acompanhou os pacientes por um período de dois anos mostrou uma associação na redução no uso do corticoide oral com o aumento da aderência ao tratamento de manutenção, ressaltando a importância das intervenções educacionais para o controle da asma.^(^ 20 ^)^

É interessante observar que o número de pacientes no Brasil (41%) que relatou possuir um plano terapêutico por escrito foi maior do que o de pacientes nos EUA (32%).^(^ 15 ^)^ No estudo AIRLA,^(^ 12 ^)^ em 2003, observou-se que a proporção de pacientes adultos (38%) que tinham um plano de ação elaborado era muito parecido com o número atual, demonstrando que, apesar das recomendações da Sociedade Brasileira de Pneumologia e Tisiologia^(^ 2 ^)^ e de GINA,^(^ 8 ^)^ não houve progresso em relação a esse tema. 

O presente estudo seguiu o mesmo delineamento utilizado no estudo AIM^(^ 16 ^)^ e foi similar ao do AIRLA.^(^ 12 ^)^ Porém, observamos algumas limitações em nosso estudo. Foram avaliadas apenas quatro cidades brasileiras no estudo, o que pode não representar a realidade brasileira geral. No entanto, é muito difícil abranger toda a população de um país em estudos com esse tipo de delineamento. Entretanto, como cada cidade incluída provém de uma região do país, essa limitação fica atenuada. Outra limitação foi o fato de não ter sido realizada espirometria, exame que também faz parte dos critérios de controle da doença. Porém, uma vez que o objetivo maior do estudo era caracterizar a realidade do paciente e seu dia a dia, a classificação do controle da doença apenas pelo questionário já foi suficiente para mostrar que essa meta ainda não foi obtida no manejo da asma. Por fim, tanto o diagnóstico da asma quanto os demais dados foram obtidos por autorrelato e não retirados de prontuários médicos.

Com esse estudo, concluímos que a grande maioria dos asmáticos brasileiros não apresenta asma adequadamente controlada segundo os critérios da GINA, e devemos atribuir esse baixo controle à baixa utilização da medicação de manutenção. Em consequência, observamos que as medicações de alivio e corticoide oral são muito frequentemente utilizadas pelos pacientes com doença parcialmente controlada ou não controlada. Portanto, maiores esforços devem ser feitos para que o tratamento da asma seja adequadamente prescrito e que a aderência ao plano terapêutico seja atingida.
